# Efficacy and Safety of Endovascular Therapy with Common Femoral Artery Endarterectomy Site Access in Patients with Lower Extremity Artery Disease

**DOI:** 10.3400/avd.oa.25-00121

**Published:** 2026-01-24

**Authors:** Shingo Mochizuki, Taira Kobayashi, Takanobu Okazaki, Kazuki Maeda, Shogo Emura, Katsutoshi Sato, Hitoshi Tachibana, Daisuke Futagami, Toshifumi Hiraoka, Risa Inoue, Tomoyasu Sato, Shinya Takahashi

**Affiliations:** 1 Department of Cardiovascular Surgery, Akane-Foundation Tsuchiya General Hospital, Hiroshima, Hiroshima, Japan; 2 Department of Cardiovascular Surgery, JA Hiroshima General Hospital, Hatsukaichi, Hiroshima, Japan; 3 Department of Cardiovascular Surgery, National Hospital Organization Higashihiroshima Medical Center, Higashihiroshima, Hiroshima, Japan; 4 Department of Cardiovascular Surgery, JA Onomichi General Hospital, Onomichi, Hiroshima, Japan; 5 Department of Cardiovascular Surgery, Hiroshima City North Medical Center Asa Citizens Hospital, Hiroshima, Hiroshima, Japan; 6 Department of Cardiovascular Surgery, Fukuyama Cardiovascular Hospital, Fukuyama, Hiroshima, Japan; 7 Department of Cardiovascular Surgery, National Hospital Organization Kure Medical Center and Chugoku Cancer Center, Kure, Hiroshima, Japan; 8 Center of Home Medical Care, Dozen Hospital, Tokyo, Japan; 9 Department of Radiology, Akane-Foundation Tsuchiya General Hospital, Hiroshima, Hiroshima, Japan; 10 Department of Cardiovascular Surgery, Hiroshima University Hospital, Hiroshima, Hiroshima, Japan

**Keywords:** common femoral artery, endarterectomy, endovascular therapy, lower extremity artery disease, access site

## Abstract

**Objectives:**

The purpose of this study was to evaluate the results of endovascular therapy (EVT) with common femoral artery (CFA) endarterectomy site access for lower extremity artery disease (LEAD).

**Methods:**

Records were reviewed retrospectively for patients who underwent EVT with CFA endarterectomy site access from 2014 to 2023 at 7 hospitals.

**Results:**

A total of 74 EVT procedures with CFA endarterectomy site access were performed in 65 patients with LEAD. The median [interquartile range] interval between CFA endarterectomy and the first EVT access was 435 [237–1153] days. Technical success of EVT was achieved in 72 procedures (97%). Technical success of the puncture was achieved in all 74 procedures (100%). The median [interquartile range] puncture time and hemostasis time were 4 [2–6] and 13 [10–20] min, respectively. Two cases (3%) had access site hematoma, which was cured with conservative treatment.

**Conclusions:**

The CFA after endarterectomy may be a safe and suitable access site for EVT.

## Introduction

Common femoral artery (CFA) endarterectomy in patients with lower extremity artery disease (LEAD) is the standard treatment for a CFA occlusive lesion.^1–5)^ However, endovascular therapy (EVT) for a femoropopliteal lesion is now performed worldwide because of technical and device advances, such as the self-expandable nitinol stent, drug-coated balloon, and drug-eluting stent. In general, a CFA lesion is more calcified than other lesions, and assessment with plain balloon angioplasty alone is ineffective. Therefore, EVT with stents may be an effective treatment for a CFA occlusive lesion.^6,7)^

In 2017, in the first randomized controlled trial of CFA endarterectomy and CFA stenting, Gouëffic et al.^8)^ demonstrated that CFA stenting had a similar durability to that of CFA endarterectomy. Surgeons are often unwilling to treat CFA occlusive lesions endovascularly with stents owing to the fear of stent fracture and of compromising the vascular access site in the future. However, the CFA after endarterectomy may be needed as a future access site. Patients who have undergone bilateral CFA endarterectomy or have an ipsilateral infrapopliteal lesion after ipsilateral CFA endarterectomy may require puncture of the CFA endarterectomy site. To our knowledge, there is only 1 study of the CFA approach after endarterectomy.^9)^ Therefore, the purpose of the current study was to evaluate the efficacy and safety of EVT with CFA endarterectomy site access.

## Materials and Methods

Between January 2014 and December 2023, we performed 74 EVT procedures with CFA endarterectomy site access for 65 patients with LEAD at 7 hospitals in Japan. EVT was defined as endovascular intervention for symptomatic patients with lower extremity artery occlusive lesions using plain old balloon angioplasty, drug-coated balloon, drug-eluting stent, or bare-nitinol stent. Patients receiving EVT with repeat CFA endarterectomy site access were excluded from the study. Patient backgrounds, procedural details, and comorbidities were evaluated. The primary endpoint of the study was the technical success of EVT and puncture. The primary safety endpoint was access site complications, and the secondary endpoints were the puncture and hemostasis times of EVT. Technical success of EVT was defined as ≤30% remaining stenosis, based on the completion angiogram. Technical success of a puncture was defined as insertion of a scheduled sheath without complications. Puncture time was defined as the interval between local anesthesia administration and sheath insertion. Hemostasis time was defined as the manual compression time.

Patient background, EVT details, and postprocedural outcomes were retrieved from the PUMPKIN (A Prospective and Retrospective, MUlti-Center PAD Study of HiroshiMa University GrouP for RisK AnalysIs of Survival and PateNcy) registry dataset. Adverse limb events were defined as untreated loss of patency of the revascularization, reintervention on the revascularized segment, or major amputation (above or below the knee) of the revascularized limb. Factors affecting the need for an additional device for sheath insertion, prolonged puncture time (≥10 min), and prolonged hemostasis time (≥20 min) were analyzed.

The study was performed in accordance with the Declaration of Helsinki. The protocols were reviewed and approved by the institutional review board (IRB) of Tsuchiya General Hospital, Hiroshima, Japan (approval no. E220328-1). The study was observational without intervention or invasiveness. Thus, the IRB waived the need for informed patient consent, and the opt-out method was alternatively used.

### Endarterectomy procedure

Procedural details and perioperative management were performed using routine methods chosen by each surgeon for patch angioplasty (used or not used), intimal layer fixation at a distal site (used or not used), and anesthesia (general or local). Eversion endarterectomy of the CFA was not performed.^10)^

### EVT procedure

CFA endarterectomy and EVT with CFA endarterectomy site access were performed at the same hospital. In 1 case, preoperative duplex ultrasound detected fluid collection above the anterior vessel wall of the CFA. Thus, we declined TEA access. All patients who underwent EVT were treated as inpatients. The CFA endarterectomy lesion was generally selected as the access site for cases of ipsilateral infrapopliteal EVT, unavailable contralateral CFA (e.g., severe calcification, severe stenosis, or post-bypass anastomosis), or post-endarterectomy for a contralateral CFA. During EVT, the selection of sheaths, guidewires, balloons, stents, and hemostatic devices was at the operator’s discretion. After administration of local anesthesia, the puncture needle was inserted into the CFA, and the guidewire was inserted through the needle. Before sheath insertion, the access site was dilated with a dilator alone in some patients.

A 4-, 5-, 6-, or 7-Fr sheath was inserted. If sheath insertion was difficult, an additional device (4-Fr sheath or hardwire) was used: the 4-Fr sheath was first inserted, and then it was replaced with a larger one. Unfractionated heparin sodium (3000 units) was administered intravenously. The guidewire was passed through the lesion, and the lesion was expanded using an optimally sized balloon. For an iliac lesion, self-expandable nitinol stents were deployed in most patients. For a femoropopliteal lesion, a drug-coated balloon was the first choice. A self-expandable nitinol stent, stent graft, or drug-eluting stent was deployed only when flow-limiting dissection or recoil occurred. For an infrapopliteal lesion, balloon angioplasty alone was performed. After the EVT procedure, manual compression of the puncture site was used in all patients. In recent cases, we used the ExoSeal vascular closure device (Cordis, Warren, NJ, USA) and the Perclose ProGlide system (Abbott Vascular, Temecula, CA, USA). The use of vascular closure devices was at the operator’s discretion.

### Medication

Periprocedural medications were at the discretion of the individual physicians. Patients who were already taking antiplatelet agents continued to take the same medicines. For patients taking no antiplatelet agents, aspirin (100 mg daily), or clopidogrel (75 mg daily), or both were started at least 1 week before EVT and continued thereafter.

### Statistical analysis

Statistical analyses were conducted using IBM SPSS Statistics for Windows (v. 20.0; IBM, Armonk, NY, USA). Continuous variables were expressed as median [interquartile range]. Categorical variables were presented as absolute values and percentages. Differences in demographics, background factors, and operative details were compared by Fisher’s exact test for categorical variables and Mann–Whitney U test for continuous variables. A P <0.05 was considered significant.

## Results

In the study period, initial EVT with CFA endarterectomy site access was performed for 79 limbs in 70 patients. Puncture times were unavailable for 5 patients, and hemostatic times were unavailable for 4 patients. Full data could not be collected in 5 cases; therefore, 74 cases (limbs) were included in the final analysis. Demographics and cardiovascular risk factors were shown in **Table 1**. The median age was 78 [70–83] years, and 88% of the patients were male. The median body mass index (BMI) was 21.4 [19.9–23.1] kg/m^2^. The patients often had diabetes (47%) and end-stage renal disease with hemodialysis (32%). The median interval between endarterectomy and initial EVT was 435 [237–1153] days. The median vessel diameter of the CFA was 9.6 [7.9–11.3] mm.

**Table:** Table 1 Patient characteristics

Variables	N (%) or median [IQR]
Patients/limbs	65/74
Age (years)	78 [70–83]
Male	65 (88)
Body mass index (kg/m^2^)	21.4 [19.9–23.1]
Body weight (kg)/body height (cm)	53 [49–60]/159[152–164]
Hypertension	67 (91)
Dyslipidemia	45 (61)
Diabetes	35 (47)
Coronary artery disease	28 (38)
Chronic heart failure	9 (12)
Atrial fibrillation	4 (5)
Cerebrovascular disease	17 (23)
Chronic kidney disease	57 (77)
Hemodialysis	24 (32)
Smoking history	53 (71)
Functional status	
Independent	57 (77)
Partially dependent	14 (19)
Dependent	3 (4)
Rutherford classification	
2/3/4/5/6	14 (19)/42(57)/5(7)/11(15)/2(3)
EA method	
Primary closure	31 (42)
Vein patch/bovine patch/artificial patch angioplasty	28 (38)/14 (19)/1 (1)
Vessel diameter (mm)	9.6 [7.9–11.3]
Interval between EA and the initial EVT (days)	435 [237–1153]
Medication	
DOAC	6 (8)
Warfarin	14 (19)
SAPT	51 (69)
DAPT	23 (31)
Statin	40 (54)

Data are presented as N (%) or median [IQR].

Chronic kidney disease was defined as stage ≥3.

N: number; IQR: interquartile range; EA: endarterectomy; DOAC: direct oral anticoagulant; SAPT: single antiplatelet therapy; DAPT: double antiplatelet therapy; EVT: endovascular therapy

EVT details and hospital outcomes are shown in **Table 2**. A femoral antegrade approach was used in 35 cases (47%). The inserted sheath sizes were 4/5/6/7 Fr in 2(3%)/1(1%)/42(88%)/6 (13%) cases. An ExoSeal vascular closure device was used in 38 cases (51%), and the Perclose ProGlide system was used in 2 cases (3%). Technical success of EVT was achieved in 72 of the 74 procedures (97%). The 2 technical failure cases were as follows: in a case with superficial femoral artery long chronic total occlusion, the wire did not cross the arteriosclerotic lesion, and a femoropopliteal bypass was used; and in a case with toe ulcer, the wire did not cross the dorsalis pedis artery, and only the proximal lesion (anterior tibial artery) could be successfully dilated, resulting in wound healing. Technical success of the puncture was achieved in all 74 procedures (100%). An additional device was used in 12 cases (16%) (4-Fr sheath in 9 cases; hard wire in 3 cases). No cases needed surgery for a pseudoaneurysm or had puncture site infection. Two cases (antegrade approach, 1 case; retrograde approach, 1 case; 3%) had access site hematoma (including 1 requiring blood infusion), and this was cured with conservative treatment.

**Table:** Table 2 Details of endovascular treatment and hospital outcomes

Variables	N (%) or median [IQR]
Femoral antegrade approach	35 (47)
Sheath size	
4 Fr	2 (3)
5 Fr	1 (1)
6 Fr	64 (86)
7 Fr	7 (9)
Puncture time (min)	4 [2–6]
Additional device for puncture	12 (16)
Small sheath	9 (12)
Hard wire	3 (4)
Treatment lesion	
Aortoiliac	27 (36)
Femoropopliteal	43 (58)
Infrapopliteal	11 (15)
Bypass graft	6 (8)
Vascular closure device	
ExoSeal	38 (51)
Perclose ProGlide	2 (3)
Hemostasis time (min)	13 [10–20]
Technical success	72 (97)
Puncture site complication	2 (3)
Postoperative hospital stay (days)	2 [2–10]
Hospital death within 30 days	2 (4)
Cardiovascular events within 30 days	1 (2)
Cerebrovascular events within 30 days	0
Adverse limb event within 30 days	
Untreated loss of patency	1
Major amputation	0
Re-intervention	0
Other complications within 30 days	1

Data are presented as N (%) or median [IQR].

Puncture site complication was defined as pseudoaneurysm, puncture site infection, or puncture site hematoma.

An adverse limb event was defined as an untreated loss of patency of the revascularization, re-intervention on the revascularized segment, or major amputation (above or below knee) of the revascularized limb.

N: number; IQR: interquartile range

Postoperative hospital stays were 2 [2–10] days. There were 2 hospital deaths within 30 days due to chronic heart failure (n = 1) and severe aortic valve stenosis (n = 1). The only cardiovascular event within 30 days was death due to aortic valve stenosis in the latter patient. In 1 case, the superficial femoral artery was occluded, but ischemic symptoms were mild. Thus, conservative treatment without revascularization was conducted. Distal embolization occurred in 1 case, and the thrombus was retrieved 8 days later.

The median puncture and hemostasis times were 4 [2–6] and 13 [10–20] min, respectively. The need for an additional device to insert a sheath was associated with a larger sheath size, male gender, higher body weight, and greater height (**Table 3**). Age, dyslipidemia, diabetes, chronic heart failure, use of a statin, and higher BMI were associated with a prolonged puncture time (≥10 min) (**Table 4**). Higher BMI (≥25.0) was not significantly associated with dyslipidemia, diabetes, chronic heart failure, or use of statins. Prolonged hemostasis time (≥20 min) was associated with chronic heart failure, use of anticoagulants, and non-use of a vascular closure device (**Table 5**). Patch material (great saphenous vein [GSV] vs. bovine) did not affect the outcomes.

**Table:** Table 3 Patient characteristics and details of endovascular treatment with or without the use of an additional device for sheath insertion

Variables	Sheath insertion without AD	Sheath insertion with AD	P value
Limbs	62	12	
Age (years)	78 [71–83]	79 [70–83]	0.99
Male	43 (69)	12 (100)	0.02
Body mass index (kg/m^2^)	21.2 [19.6–23.4]	22.4 [20.1–22.8]	0.39
Body weight (kg)	52 [47–59]	57 [54–62]	0.02
Body height (cm)	157 [151–164]	163 [160–164]	0.04
Hypertension	56 (90)	11 (92)	0.63
Dyslipidemia	37 (60)	8 (67)	0.46
Diabetes	31 (50)	4 (33)	0.29
Coronary artery disease	25 (40)	3 (25)	0.25
Chronic heart failure	6 (10)	3 (25)	0.16
Atrial fibrillation	4 (6)	0 (0)	0.49
Cerebrovascular disease	14 (23)	3 (25)	0.56
Chronic kidney disease	48 (77)	9 (75)	0.56
Hemodialysis	19 (31)	5 (42)	0.33
Smoking history	44 (71)	9 (75)	0.54
Medication			
Anticoagulant	16 (26)	4 (33)	0.41
Single antiplatelet therapy	44 (71)	7 (58)	0.29
Double antiplatelet therapy	18 (29)	5 (42)	0.29
Statin	31 (52)	8 (67)	0.34
EA with patch angioplasty	36 (58)	7 (58)	0.99
Interval between EA and EVT (days)	432 [229–1153]	726 [271–1087]	0.70
Vessel diameter (mm)	9.0 [7.9–11.3]	10.0 [7.9–11.8]	0.65
Sheath size			
4, 5, and 6 Fr	60 (97)	8 (75)	0.01
7 Fr	2 (3)	4 (25)	
Femoral antegrade approach	31 (50)	8 (75)	0.29

Data are presented as N (%) or median [interquartile range].

EA: endarterectomy; EVT: endovascular treatment; AD: additional device

**Table:** Table 4 Patient characteristics and details of endovascular treatment with or without a prolonged puncture time (>10 min)

Variables	Early (≤10 min)	Prolonged (>10 min)	P value
Limbs	64	10	
Age (years)	81 [79–85]	76 [69–82]	0.03
Male	47 (73)	8 (80)	1.00
Body mass index (kg/m^2^)	21.0 [19.9–22.7]	23.1 [22.5–24.0]	0.03
Body weight (kg)	53 [48–59]	57 [50–59]	0.41
Body height (cm)	159 [153–165]	158 [153–161]	0.31
Hypertension	57 (89)	10 (100)	0.58
Dyslipidemia	36 (56)	9 (90)	0.04
Diabetes	27 (42)	8 (80)	0.03
Coronary artery disease	24 (38)	4 (40)	0.57
Chronic heart failure	5 (8)	4 (40)	0.02
Atrial fibrillation	3 (5)	1 (10)	0.45
Cerebrovascular disease	14 (22)	3 (30)	0.41
Chronic kidney disease	47 (73)	10 (100)	0.06
Hemodialysis	21 (33)	3 (30)	0.59
Smoking history	44 (69)	9 (90)	0.16
Medication			
Anticoagulant	17 (42)	3 (30)	0.54
Single antiplatelet therapy	44 (69)	7 (70)	0.63
Double antiplatelet therapy	20 (31)	3 (30)	0.63
Statin	31 (48)	9 (90)	0.01
EA with patch angioplasty	39 (61)	4 (40)	0.18
Interval between EA and EVT (days)	567 [245–1168]	389 [179–680]	0.56
Vessel diameter (mm)	9.3 [8.0–11.6]	10.0 [8.1–10.9]	0.82
Sheath size			
4, 5, and 6	60 (94)	8 (80)	0.18
7 Fr	4 (6)	2 (20)	
AD for puncture	8 (13)	3 (30)	0.16
Femoral antegrade approach	35 (55)	4 (40)	0.35

Data are presented as N (%) or median [interquartile range].

EA: endarterectomy; EVT: endovascular treatment; AD: additional device

**Table:** Table 5 Patient characteristics and details of endovascular treatment with or without a prolonged hemostasis time (>20 min)

Variables	Early (≤20 min)	Prolonged (>20 min)	P value
Limbs	62	12	0.35
Age (years)	79 [69–83]	78 [77–82]	0.59
Male	45 (73)	10 (83)	0.35
Body mass index (kg/m^2^)	21.3 [19.9–23.0]	22.4 [19.3–23.6]	0.79
Body weight (kg)	53 [49–59]	56 [48–64]	0.62
Body height (cm)	159 [152–164]	161 [158–164]	0.42
Hypertension	55 (89)	12 (100)	0.27
Dyslipidemia	39 (63)	6 (50)	0.30
Diabetes	28 (45)	7 (58)	0.40
Coronary artery disease	24 (39)	4 (33)	0.50
Chronic heart failure	5 (8)	4 (33)	0.03
Atrial fibrillation	3 (5)	1 (8)	0.52
Cerebrovascular disease	14 (23)	3 (25)	0.56
Chronic kidney disease	46 (74)	11 (92)	0.18
Hemodialysis	19 (31)	5 (42)	0.33
Smoking history	44 (71)	3 (25)	0.54
Medication			
Anticoagulant	12 (19)	8 (75)	0.002
Single antiplatelet therapy	43 (69)	4 (33)	0.55
Double antiplatelet therapy	19 (31)	8(67)	0.55
Statin	34 (55)	6 (50)	0.76
EA with patch angioplasty	36 (58)	7 (58)	0.99
Interval between EA and EVT (days)	568 [259–1189]	296 [96–881]	0.10
Vessel diameter (mm)	10.0 [8.1–11.9]	8.6 [7.7–10.0]	0.19
Sheath size			
4, 5, and 6 Fr	57 (92)	11 (92)	0.67
7 Fr	5 (8)	1 (8)	0.67
AD for puncture	7 (11)	4 (33)	0.71
Antegrade approach	33 (53)	6 (50)	0.84
Puncture time (min)	4 [2–6]	4 [3–6]	0.59
Vascular closure device use	38 (61)	2 (17)	0.005

Data are presented as N (%) or median [interquartile range].

EA: endarterectomy; EVT: endovascular treatment; AD: additional device

## Discussion

The 3 main results of this study were as follows. First, the technical success rate of EVT was 97% and that of sheath insertion was 100%, which was acceptable. Second, the median puncture time was 4 [2–6] min, which was favorable. Third, the median hemostasis time was 13 [10–20] min, and complications at the access site occurred in only 2 cases (3%). We note that EVT was performed for various lesion types and with various devices; thus, the satisfactory success rate of 97% suggests that the use of a CFA endarterectomy site access does not adversely affect procedural success.

In the current series, 12 cases (16%) required additional devices. An operative scar with hard tissue through which inserting a puncture needle or sheath is difficult is a potential problem in creating a puncture for a CFA endarterectomy lesion.^9)^ In a study of 40 cases of EVT with CFA endarterectomy site access, 10 (25%) required an additional device for insertion of a scheduled sheath, which was similar to our findings.^9)^ In the previous series, the technical success rate of sheath insertion was 98%, also similar to the rate in our series, and the rate of difficult sheath insertions peaked from 6 months to 1 year after endarterectomy and then gradually decreased. In contrast, in our series, the interval after endarterectomy did not affect the need for an additional device.

The puncture time after endarterectomy has not been previously reported. In our series, the median puncture time of 4 [2–6] min was acceptable. Factors associated with prolonged puncture times (≥10 min) were age, dyslipidemia, diabetes, chronic heart failure, use of statins, and higher BMI. These risk factors suggest that the longer distance from the puncture site to the vessel wall in obese patients may make puncturing the vessel more difficult.

The median hemostasis time of 13 [10–20] min was favorable. Factors associated with a prolonged hemostasis time (≥20 min) were chronic heart failure, use of anticoagulants, and non-use of a vascular closure device. Various reports have shown that anticoagulation is a risk factor for puncture site complications, and oral anticoagulants may prolong hemostasis time and increase the risk of bleeding.^11–13)^ Many investigators have reported the effectiveness of using a vascular closure device in patients undergoing EVT for LEAD.^14–16)^ However, whether a vascular closure system can be applied to a femoral endarterectomy site access is controversial. In this study, an ExoSeal vascular closure device was used in 38 cases (51%). No patient required surgical treatment for a pseudoaneurysm or puncture site bleeding, which indicates the safety and efficacy of using an ExoSeal vascular closure system for femoral endarterectomy site access.

This study has several limitations, including the retrospective design, relatively small sample size, and performance of EVT for different types of lesions. Efficacy and safety could not be evaluated for each type of lesion due to the small sample size. Despite these limitations, we believe that our results provide new insights into EVT with CFA endarterectomy site access.

## Conclusion

EVT with CFA endarterectomy site access had a high technical success rate with few access site complications. These findings suggest that the CFA after endarterectomy is a safe and suitable access site for EVT.

## Declarations

### Data availability

Data will be made available by the corresponding author upon reasonable request.
